# Supplementary material for: The Systems Biology Research Tool: evolvable open-source software
Source: BMC Syst Biol. 2008 Jun 29;2:55. doi: 10.1186/1752-0509-2-55 (PMC2446383; doi:10.1186/1752-0509-2-55)
Supplement: Additional file 1 — SBRT Archive. An archive of the current version of the Systems Biology Research Tool. [file 1752-0509-2-55-S1.zip › sbrt-1.4.0/doc/developers_guide/api/sbrt/kernel/math/geom/ParallelHyperplanes.html]

ParallelHyperplanes


|  |  |  |
| --- | --- | --- |
| **Overview** \| **Package** \| **Class** \| **Use** \| **Tree** \| **Deprecated** \| **Index** \| **Help** | |  |
| **PREV CLASS**   NEXT CLASS | **FRAMES**    **NO FRAMES**     **All Classes** |  |
| SUMMARY: NESTED \| FIELD \| CONSTR \| METHOD | DETAIL: FIELD \| CONSTR \| METHOD |  |


---


## sbrt.kernel.math.geom Class ParallelHyperplanes<V>

```
java.lang.Object
  sbrt.kernel.math.geom.ParallelHyperplanes<V>
```

**Type Parameters:**: `V` - the variable type.

---

``` public class ParallelHyperplanes<V> extends java.lang.Object ```

This class is used to represent a pair of bounding parallel hyperplanes.
They have the general form: lower bound ≤ hyperplane ≤ upper bound.
These are also called half-spaces.

**Author:**
:   This class was written and documented by
    Jeremiah Wright while in the Wagner lab.

---

**Constructor Summary**

`ParallelHyperplanes(LinearComb<V> hyperplane,
Interval bounds)`
  
          Constructs a new pair of bounding parallel hyperplanes.


| **Method Summary** | |
| --- | --- |
| `boolean` | `equals(java.lang.Object obj)`             Indicates if the provided object is equal to this pair of bounding parallelt hyperplanes. |
| `Interval` | `getBounds()`             Returns the bounding values used to define the inequality. |
| `LinearComb<V>` | `getPlane()`             Returns the hyperplane. |
| `int` | `hashCode()`             Returns a content-based hash code. |
| `java.lang.String` | `toString()`             Returns a string representation of this pair of bounding parallel hyperplanes. |

**Methods inherited from class java.lang.Object**

`clone, finalize, getClass, notify, notifyAll, wait, wait, wait`

**Constructor Detail**

### ParallelHyperplanes

```
public ParallelHyperplanes(LinearComb<V> hyperplane,
                           Interval bounds)
```

:   Constructs a new pair of bounding parallel hyperplanes.

    **Parameters:**: `hyperplane` - the hyperplane.: `bounds` - the bounding values used to define the inequality.


**Method Detail**

### getPlane

```
public LinearComb<V> getPlane()
```

:   Returns the hyperplane.

    :   **Returns:**: the hyperplane.

---


### getBounds

```
public Interval getBounds()
```

:   Returns the bounding values used to define the inequality.

    :   **Returns:**: the bounding values used to define the inequality.

---


### hashCode

```
public int hashCode()
```

:   Returns a content-based hash code.

    :   **Overrides:**: `hashCode` in class `java.lang.Object`
    :   **Returns:**: a content-based hash code.

---


### toString

```
public java.lang.String toString()
```

:   Returns a string representation of this pair of bounding parallel
    hyperplanes.

    :   **Overrides:**: `toString` in class `java.lang.Object`
    :   **Returns:**: a string representation of this pair of bounding parallel hyperplanes.

---


### equals

```
public boolean equals(java.lang.Object obj)
```

:   Indicates if the provided object is equal to this pair of
    bounding parallelt hyperplanes. Two pairs of bounding parallel
    hyperplanes are equal if their bounds and hyperplanes are equal.

    :   **Overrides:**: `equals` in class `java.lang.Object`
    :   **Parameters:**: `obj` - the object with which to compare. **Returns:**: `true` if this object is equal to the provided object; `false` otherwise.


---


|  |  |  |
| --- | --- | --- |
| **Overview** \| **Package** \| **Class** \| **Use** \| **Tree** \| **Deprecated** \| **Index** \| **Help** | |  |
| **PREV CLASS**   NEXT CLASS | **FRAMES**    **NO FRAMES**     **All Classes** |  |
| SUMMARY: NESTED \| FIELD \| CONSTR \| METHOD | DETAIL: FIELD \| CONSTR \| METHOD |  |


---
